# Evaluation of Chemical and Biological Products for Control of Crown Gall on Rose

**DOI:** 10.3390/pathogens13080708

**Published:** 2024-08-21

**Authors:** Cansu Oksel, Prabha Liyanapathiranage, Madhav Parajuli, Farhat A. Avin, Christina Jennings, Terri Simmons, Fulya Baysal-Gurel

**Affiliations:** Department of Agricultural Sciences and Engineering, College of Agriculture, Otis L. Floyd Nursery Research Center, Tennessee State University, McMinnville, TN 37110, USA; coksel@tnstate.edu (C.O.); prabha.liyanapathiranage@tn.gov (P.L.); madhav.parajuli@usda.gov (M.P.); aavin@tnstate.edu (F.A.A.); cjennin7@tnstate.edu (C.J.); tsimmons15@tnstate.edu (T.S.)

**Keywords:** *Agrobacterium tumefaciens*, disease management, *Rosa* spp., woody ornamental

## Abstract

Crown gall is a soil-borne bacterial disease caused by *Agrobacterium tumefaciens*, leading to significant economic losses in many plant species. For the assessment of the biological and chemical products on crown gall, each plant’s crown region and roots were wounded, and then were dipped into their respective treatments. After the treatments, the plants were inoculated with a suspension of pathogenic *A. tumefaciens* isolate FBG1034 and maintained in a greenhouse for six months to assess them for gall formation. A quantitative real-time PCR assay was performed to quantify the *A*. *tumefaciens* using the *chvE* gene. Biological products such as the *Agrobacterium radiobacter* strain K1026, and strains 1 and 2, resulted in the lowest average root gall diameter and significantly reduced the crown gall diameter to stem diameter ratio, and the chemical product copper octanoate reduced the number of crown and root galls as well as the crown and root gall diameter compared to the inoculated, non-treated control. Moreover, both the *A. radiobacter* strain K1026 and strain 1 treatments resulted in an approximately 85% and 65% reduction in crown and root gall incidence, respectively, in both of the trials compared to the inoculated, non-treated plants. The findings of this study indicate that the use of biological and chemical products could help to suppress crown and root gall disease in rose plants.

## 1. Introduction

Roses (*Rosa* sp.), belonging to the Rosaceae family, are the most cultivated ornamental plants based on their beautiful flowers [1,2]. They rank among the top five ornamental plants worldwide [3]. In the United States, the sale of roses in 2019 generated USD 200 million from the production of 39 million plants by 1729 operations [4]. However, the production of roses is limited by several plant pathogens. *Agrobacterium tumefaciens* (syn: *Rhizobium radiobacter*) is a soil-borne pathogen that poses a threat to the yield and quality of roses [5]. *A. tumefaciens* can survive in the soil, the rhizosphere of many plants, and other natural environments [6,7]. Even under non-nursery conditions, crown gall has caused a 60% decrease in rose cut flower production in southern France, where intensive soil-less cultivation conditions were employed [8]. It can persist in symptomless rose plants and is a major cause for the spread of crown gall diseases, resulting in economic losses [9]. The pathogen has been extensively studied by researchers, as it is a significant factor in endangering the health of roses, causing poor rooting, a decrease in number of shoot formations, and the yield loss of cut flowers, affecting their commercial production [10,11,12].

The pathogenic *A. tumefaciens* has megaplasmids known as Ti (tumor-inducing plasmids) [11,13,14]. The bacteria transfer a fragment of the Ti plasmid (T-DNA) into the plant cell, where it is incorporated into the plant genome. The vir (virulence) region is responsible for plasmid transmission to the plant cell and T-DNA integration. T-DNA, or transferred DNA contains genes that are responsible for the synthesis of opines and for controlling the biosynthesis of phytohormones in host plant cells. The expression of T-DNA genes causes uncontrolled plant cell division [15,16,17]. As a result, the pathogen causes gall on the stem, crown, and root [18,19,20,21], and infects over 600 species of plants, including roses [22,23]. Different *Agrobacterium* strains are grouped based on their ability to utilize different types of opines, including agrocinopine, nopaline, and octopine [24]. The taxonomic status of *Agrobacterium* spp. has been divided into three biovars [25]. Biovar 1 includes tumorigenic *A. tumefaciens* strains harboring tumor-inducing (Ti) plasmids [26], biovar 2 is the *A. rhizogenes* strain, which harbors root-inducing (Ri) plasmids [27], and biovar 3 is the *A. vitis* strain, which harbors vitopine-type tumor-inducing (Ti) plasmids [28]. Later studies showed that *A. tumefaciens* (i.e., biovar 1) is not a single species, but rather represents a genomic species, numbered from G1 to G9 and G13. These species are collectively named the “*A. tumefaciens* species complex” [5,29,30]. *A. tumefaciens* biovar 1 is one of the Rhizobiaceae family’s most important soil-borne bacterial species [31].

The pathogen is identified using conventional disease diagnosis methods based on the isolation of this bacterium on selective or general media, and pathogenicity tests on alternative plants, such as carrots or tomatoes [32,33,34,35], as well as molecular methods like using primers designed from virulence (*vir*) genes or T-DNA sequences to detect and identify the pathogen from cultures, soils, and plant tissues [34]. Previous research indicates that *A. tumefaciens* can be detected in plant or soil samples using qPCR to target the *cheV* gene. This protein is encoded by the *chvE* gene and is responsible for a sugar-induced increase in the expression of *vir* genes [11,36,37,38].

The most effective and sustainable approach to plant disease management involves an integrated management system that includes the use of plant host resistance, less susceptible cultivars, intervention with chemical and/or biological controls, and sanitation and cultural practices aimed at reducing inoculum [39,40]. Using non-infested soil, inoculum-free plant material, and sanitized cutting tools are important steps in controlling and preventing crown gall. For this reason, pre-plant soil fumigation such as methyl bromide, chloropicrin, iodomethane, dazomet, and metam sodium in nurseries and orchards was successfully used as the primary management strategy [41]. A study reported that sanitizing cutting tools lowered gall formation in *Datura stramonium* caused by *A. tumefaciens* [38]. However, some studies have indicated that, even if all of the plant material with visible crown galls is removed from a nursery, growers may still purchase and plant apparently healthy plants that are actually infected without showing any symptoms, because the pathogen can still be present in the plants. In this way, pathogens can easily spread [7], which might cause difficulties in managing crown gall. Previously, some chemicals like 2-methoxyethyl mercury chloride, copper oxychloride, and an aqueous solution of cuprammonium were tested as pre-planting dips for apple seedlings, and copper oxychloride and an aqueous solution of cuprammonium were shown to offer good possibilities for the control of crown gall in apple seedlings [42]. Similarly, Utkhede and Smith [43] found that using copper oxychloride as a root-dip treatment effectively reduced crown gall infection in apple trees, but also resulted in phytotoxicity issues. In the other study, copper oxychloride was used to control crown gall disease on roses, but it was not successful in inhibiting crown gall growth [44]. Biological control agents are known to play a critical and successful role in the management of crown gall. *Agrobacterium tumefaciens* has been successfully controlled using the *A. radiobacter* (*R. rhizogenes*) K84 and K1026 strains [45]. The mechanism of this control is the production by an avirulent *A. radiobacter* strain, K84, of a fraudulent adenine nucleotide analog called agrocin 84 [46]. Agrocin 84 acts like an antibiotic against *A. tumefaciens*. So, the *A. radiobacter* strain K84 inhibits *A. tumefaciens* while both of them compete for colonization in the root and wound sites of the plant [47,48]. However, the use of the *A. radiobacter* strain K84 showed a failure to control agrocin 84 production, resulting in the loss of biocontrol [48,49,50]. Recombination DNA techniques have been used to construct a new biological strain, K1026, identical to the K84 strain. The K1026 strain does not have the ability to transfer its mutant agrocin 84 plasmid to other bacteria [51,52]. The *A. radiobacter* strain K1026 was suggested as a safer organism than the K84 strain to control crown gall [53]. Several studies have indicated that other bacterial strains, including *Rahnella aquatilis* HX2 [54], *Pseudomonas fluorescens*, *Bacillus subtillis* [55], *Curtobacterium* sp. [56], *Pseudomonas* sp. Sn48, *Pseudomonas* sp. Ba35, *Pantoea* sp. Sa14 [57], and *B. velezensis* CLA178 [11], have various effects in reducing crown gall development. No chemical products have been effectively suggested for the control of crown gall disease. The biological control of crown gall disease using the *A. radiobacter* strains K84 and K1026 is a significant achievement in helping propagation nurseries battle with this disease worldwide. However, alternative products should be researched as chemical products used to control crown gall whenever serious failures or unexpected deleterious effects of the suggested biological control products are considered. The objective of this study was to evaluate the efficacy of chemical agents in preventing crown gall disease in roses. Furthermore, considering the comparative data available, it is recommended that *A. radiobacter* strains continue to be utilized as an effective biological solution for controlling crown gall.

## 2. Materials and Methods

### 2.1. Isolation and Identification of Agrobacterium tumefaciens

‘Pink Knock Out^®^’ rose plants displaying crown galls were received from a commercial nursery in McMinnville, TN, USA. The galls were washed with running tap water and dried. Small sections of the surface tissues were removed using a sterile scalpel. The tissues were then sterilized with sodium hypochlorite (0.525%) for 1 min and washed three times with sterilized water. The galls were chopped and macerated in sterile saline buffer (5% NaCl). The suspensions were plated onto King’s B (KB) and 1A media, and incubated for a photoperiod of 8 h of light at 25 °C. After the incubation, single colonies were picked and re-streaked to ensure purity, and then used for the identification. Biochemical tests were performed using the methods described by Kovacs [58] and Lelliott and Stead [23]. The isolates were tested for the Gram stain, oxidase and catalase reaction, and starch and gelatin hydrolysis. Total genomic DNA was extracted from the isolates grown on 1A medium for 2 days at 25 °C using the Qiagen PowerLyzer Ultraclean Microbial DNA Isolation Kit (Qiagen, Germantown, MD, USA). The DNA concentration was verified with a NanoDrop spectrophotometer (Thermo Fisher Scientific, Waltham, MA, USA). The bacterial isolates were identified using the PCR primers 8F/1492R to amplify the 16S ribosomal DNA. The detection was followed by amplification using the primer pair virD2A/virD2C, which can amplify a 226 bp fragment in all tumorigenic *Agrobacterium* species [59], and VCF/VCR for the identification of the *A. tumefaciens* biovar 1 species complex [60]. The primers ACC-F/ACC-R [24] and RB-F/RB-R [61] were used to detect agrocinopine and nopaline so to characterize the isolates regarding the utilization of the carbon sources. The PCR products were separated in a 1% agarose gel, and bands were visualized under a Gel Doc EZ imager (Bio-Rad, Hercules, CA, USA). The 16S ribosomal DNA amplicons were purified using the Wizard SV Gel and PCR Clean-Up system (Promega, Madison, WI, USA), and then sent to GenHunter Corporation (Nashville, TN, USA) for sequencing. The resulting sequences were deposited in the NCBI GenBank.

### 2.2. Pathogenicity Test on Carrot Slices

A pathogenicity test was conducted on carrot slices using previously established protocols [62,63]. Briefly, carrots were surface-sterilized and cut into small slices. They were then placed in Petri dishes with sterile, moist filter paper, and a bacterial suspension (1 × 10^9^ CFU mL^−1^) was spread across each carrot slice. The plates were kept at room temperature in the dark, and the filter papers were periodically moistened [35]. The pathogenicity test was assessed after 3 weeks. As a control, carrot slices were treated with sterile distilled water. The assay was performed twice with three replicates. Gall development was observed and compared with the control after inoculation.

### 2.3. Efficacy of Chemical and Biological Product Treatments on Control of Crown Gall on Rose

#### 2.3.1. Inoculum Preparation

The isolate of *A. tumefaciens* (FBG1034, GenBank accession no: OR838799), identified through biochemical tests, molecular analyses, and pathogenicity tests, was randomly selected among the *A. tumefaciens* isolates and used for the chemical and biological product treatments to control the crown gall on rose. Two-day-old cultures of *A. tumefaciens* grown on 1A medium at 25 °C were suspended in 1 L of sterile distilled water, and the concentration was adjusted to 1 × 10^9^ CFU mL^−1^ (OD: 0.2) to prepare the inoculum.

#### 2.3.2. Treatments

The treatments included the *A. radiobacter* strain K1026 (Nogall; Evergreen Growers Supply, LLC., Clackamas, OR, USA), *A. radiobacter* strain 1 and strain 2 (Galltrol A strains 1 and 2: AgBioChem Inc., Los Molinos, CA, USA) as biological products, and didecyl dimethyl ammonium chloride (KleenGrow; PACE 49 Inc., Delta, BC, Canada), copper octanoate (Camelot O; SePRO, Rocky Mount, NC, USA), and a combination of didecyl dimethyl ammonium chloride + copper octanoate (KleenGrow + Camelot O) as chemical products (Table 1).

#### 2.3.3. Experimental Design

The greenhouse experiments were conducted at the Tennessee State University Otis L. Floyd Nursery Research Center in McMinnville, TN, USA. One-year-old rooted cuttings of rose ‘Radcon’ Pink Knock Out^®^ plants were received from a commercial nursery. Plants were monitored for any symptoms and then potted in number 1 nursery containers (16-cm diameter × 16-cm height; Hummert International, Earth City, MO, USA) filled with Morton’s Nursery mix (processed pine bark (55–65%), Canadian sphagnum peat, and sand) on 1 May 2022 (Morton’s Horticultural Products, McMinnville, TN, USA). Plants were watered for 2 min twice daily using an overhead automatic irrigation system (SpinNet nozzle; Hummert International, Earth City, MO, USA). Plants were fertilized with 5 g of 18-6-8 Nutricote controlled-release granular fertilizer (Florikan E.S.A. LLC., Sarasota, FL, USA). Then, 7.5 g of Miracle-Gro^®^ Water Soluble All-Purpose Plant Food (Scoot’s Miracle-Gro Products, Inc., Marysville, OH, USA) was mixed in 1 L of water and applied to the rose plants (100 mL for each plant) on 14 May 2022. Two experiments were conducted from 3 June to 8 December 2022 (trial 1) and from 11 October 2022 to 13 April 2023 (trial 2). Ten single-plant replicates per treatment were arranged in a completely randomized design. The method developed by Rhouma et al. [64] was modified to treat the rooted rose cuttings. The plants were then taken out of their pots, and the soil was gently shaken off the roots using tap water. Each plant was wounded once at the crown region above the potting mix surface by making a 1 cm lateral cut using a sterilized blade; following that, the roots were trimmed at six sites using sterile scissors. Then, the plants were individually dipped into their respective biological and chemical products for 15 min. After the treatments, the plants were replanted in the same pots and artificially inoculated with the *A. tumefaciens* suspension (1 × 10^9^ CFU mL^−1^) at the rate of 150 mL/plant on 3 June 2022 (trial 1) and 11 October 2022 (trial 2). Non-treated, non-inoculated plants and non-treated, inoculated plants served as the negative and positive controls, respectively. The average maximum temperatures in the greenhouse for June, July, August, September, October, November, and December 2022, and January, February, March, and April 2023, were 33.9, 30.2, 28.8, 28.2, 27.7, 27.6, 25.5, 26.9, 27, 27.3, and 27.3 °C, while the average minimum temperatures were 19, 20.3, 19.7, 17.9, 17.5, 14.4, 15.4, 21.1, 22.2, 22.8, and 23.05 °C, respectively. The average maximum relative humidity in the greenhouse for June, July, August, September, October, November, and December 2022, and January, February, March, and April 2023, was 100%, and the average minimum relative humidity was 84.5, 61.4, 81.9, 78.5, 82.5, 84.5, 91.3, 92.0, 74.2, 73.8, and 71.2%, respectively.

#### 2.3.4. Recording Plant Growth and Crown and Root Gall Disease

The initial plant height and width were measured on 3 June 2022 (trial 1) and 11 October 2022 (trial 2). The final plant height and width, plant total fresh weight, root fresh weight, defoliation, chlorophyll content, crown gall diameter, and root gall diameter were recorded on 8 December 2022 (trial 1) and 13 April 2023 (trial 2), respectively. The height was measured from the base of the stem at the substrate level to the top of the highest terminal bud on the main stem. The plant width was the average of the widest horizontal spread, from leaf tip to leaf tip, and the spread perpendicular to the widest spread. The increase in the plant height and width was calculated by subtracting the initial height and width from the final height and width [65]. Defoliation was assessed using the following formula: ((number of nodes without leaves)/(total number of nodes) × 100) [66]. The chlorophyll content (SPAD value) of the fully expanded leaves was measured with a SPAD-502 leaf chlorophyll meter (Spectrum Technologies Inc., Aurora, IL, USA).

To evaluate the chemical and biological product efficiency on the crown and root galls, the crown gall and root gall diameters were measured horizontally and vertically using a Spurtar Vernier caliper (Rexbeti, Auburn, WA, USA), and the average diameter values were used in the analysis. The number of root galls and crown galls was recorded for each plant on 8 December 2022 (trial 1) and 13 April 2023 (trial 2). The disease incidence was estimated for each treatment as the number of symptomatic plants among 10 plants [56]. The ratios of the root gall diameter to the root diameter (RGD/RD) and the crown gall diameter to the stem diameter (GD/SD) were calculated [67,68].

### 2.4. Quantification of Agrobacterium tumefaciens from Rose Roots Using qPCR

To determine the amount of *A. tumefaciens* in the roses treated with the *A. radiobacter* strain K1026 (Nogall), *A. radiobacter* strain 1 and strain 2 (Galltrol A strains 1 and 2), didecyl dimethyl ammonium chloride (KleenGrow), copper octanoate (Camelot O), and the combination of didecyl dimethyl ammonium chloride + copper octanoate (KleenGrow + Camelot O), a qPCR was performed on DNA samples obtained by randomly cutting the roots analyzed during trial 2. At the end of trial 2, the roots from each rose were randomly cut into 0.5 to 1 cm pieces using sterile scissors and then transferred into a sterile 1.5 mL tube. The analysis was also conducted on inoculated, non-treated and non-inoculated, non-treated rose control plants. The total DNA was extracted using the Qiagen PowerLyzer Ultraclean Microbial DNA Isolation Kit (Qiagen, Germantown, MD, USA). The concentration of all of the DNA samples was quantified and maintained at 5 ng/μL of DNA. Each qPCR reaction contained 10 μL of GoTaq qPCR Master Mix (Promega, Madison, WI, USA), 2 μL (10 μM) of the *chvE* gene of forward (5′-GCT GTC CCA GAT CGA AAA-3′) and reverse (5′-GCC TGC TTC AGA ACG TC-3′) primers [36], 2 μL of the template DNA, and 4 μL of nuclease-free water to make the final reaction volume of 20 μL. In the negative control reactions, 2 μL of nuclease-free water replaced the DNA in the same mixtures. The qPCR was performed using the Bio-Rad CFX96 Touch Real-Time PCR Detection System (Bio-Rad, Hercules, CA, USA). The following real-time PCR program settings were used: 5 min at 95 °C, 40 cycles of 15 s at 95 °C, and 30 s at 55 °C. After the final amplification cycle in the qPCR, a standard melt curve analysis was performed by initially heating the samples to 95 °C and then cooling them to 65 °C. The samples were then gradually reheated to 95 °C, with the temperature increasing by 0.5 °C every 5 s. A standard curve plotted the logarithm of the 10-fold serial dilutions from 22.6 ng/µL to 22.6 fg/µL of pure *A. tumefaciens* DNA against the cycle threshold (Ct) values. All of the qPCR assays were replicated three times.

### 2.5. Statistical Analysis

The plant height, width, total plant fresh weight, root fresh weight, crown gall diameter, root gall diameter, number of root and crown galls, defoliation, chlorophyll content (SPAD value), and DNA concentration for the qPCR data were compared among the treatments using a one-way ANOVA in SAS 9.4 (SAS Institute, Cary, NC, USA). The post hoc Fisher’s least significant difference test was performed to compare the means (α = 0.05) when the effects were significant. The total number of crown and root galls formed, crown and root gall diameters, and the ratio of the root gall diameter to the root diameter (RGD/RD) and the crown gall diameter to the stem diameter (GD/SD) were analyzed using GENMOD fitted in negative binomial. The crown and root gall diameter values were analyzed using a mixed model (PROC GLIMMIX) fitted in the beta distribution in SAS 9.4. Pearson’s product–moment correlation using R software version 4.2.3 was used to assess the relationship between the plant growth parameters and the number and diameter of root and crown galls.

## 3. Results

### 3.1. Isolation and Identification of Agrobacterium tumefaciens

**Figure 1:**
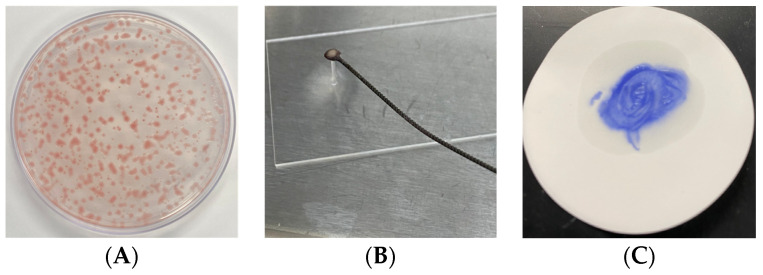

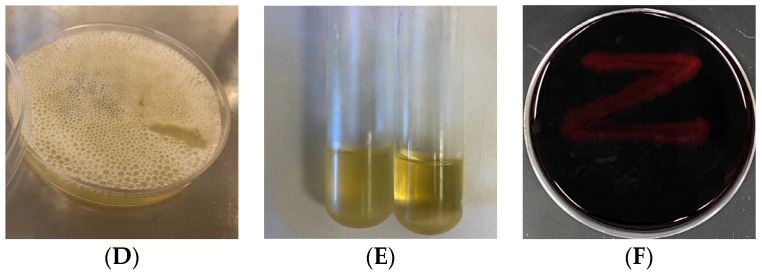
Identification of the tumorigenic *A. tumefaciens* isolates FBG1034 and FBG1035 using biochemical tests: (**A**) pink to pinkish-red color colony on 1A medium; (**B**) Gram-negative bacteria based on the Gram staining; (**C**) in the oxidase test, the inoculated filter paper turned violet immediately after adding the reagent; (**D**) in the catalase test, bubbles were formed on the Petri plate after adding H_2_O_2_; (**E**) the isolates were not produced gelatinases; (**F**) the isolates were unable to hydrolyze starch.

The colonies of the isolates FBG1034 and FBG1035 were observed to be pink to pinkish red in color, circular, and rod-shaped on 1A medium. Moreover, the colonies were circular, mucoid, pearly white, and non-fluorescent on KB medium after three days of incubation. The isolates were Gram-negative, and the biochemical test results were positive for catalase and oxidase, but negative for gelatin and starch (Figure 1). Based on the phenotypic and biochemical test results, the isolates were identified as the *A. tumefaciens* species complex biovar 1 [69].

**Figure 2:**
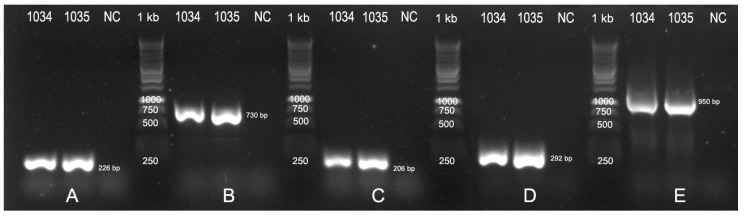
Identification of the tumorigenic *A. tumefaciens* isolates FBG1034 and FBG1035 using the PCR amplification of specific and universal genetic markers: (**A**) specific genus level to all tumorigenic *Agrobacterium* species (primers: virD2A/virD2C); (**B**) specific genus level to the *A. tumefaciens* biovar 1 species complex (primers: VCF/VCR); (**C**) specific genus level to the nopaline *A. tumefaciens* type (primers: RB-F/RB-R); (**D**) specific genus level to the agrocinopine *A. tumefaciens* type (primers: ACC-F/ACC-R); (**E**) ribosomal 16S universal primers (8F/1492R).

The isolates produced a 224 bp amplicon using the primer pair VirD2A/VirD2C, which is specific to all tumorigenic *Agrobacterium* species. Additionally, they produced a 730 bp amplicon with the VCF/VCR primer pair, which is specific to the *A. tumefaciens* biovar 1 species complex [70,71], a 206 bp amplicon with the RB-F/RB-R primer pair, which is specific to nopaline, and a 292 bp amplicon with the ACC-F/ACC-R primer pair, which is specific to agrocinopine, after the PCR analysis (Figure 2). The NCBI BLASTn analysis of the 16S ribosomal DNA gene sequences (GenBank accession nos. FBG1034: OR838799 and FBG1035: OR838800) showed a 100% identity to the sequence of *A. tumefaciens* (GenBank accession no. FR828334).

### 3.2. Pathogenicity Test on Carrot Slices

**Figure 3:**
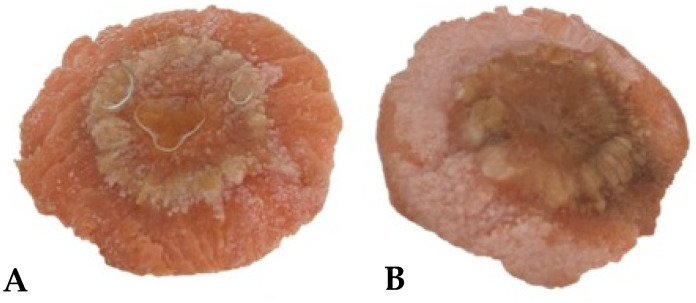
The formation of galls on the carrot slices caused by the *A. tumefaciens* isolates FBG1034 (**A**) and FBG1035 (**B**).

The test was conducted to confirm whether the isolates inoculated in the carrot slices initiated the formation of galls (Figure 3). The results showed that gall formation was exhibited on the carrot slices two weeks after the FBG1034 and FBG1035 inoculation. There were no differences observed in the gall formation between the isolates. No symptoms were observed on the negative control.

### 3.3. Efficacy of Chemical and Biological Product on Control of Crown Gall on Rose

**Figure 4:**
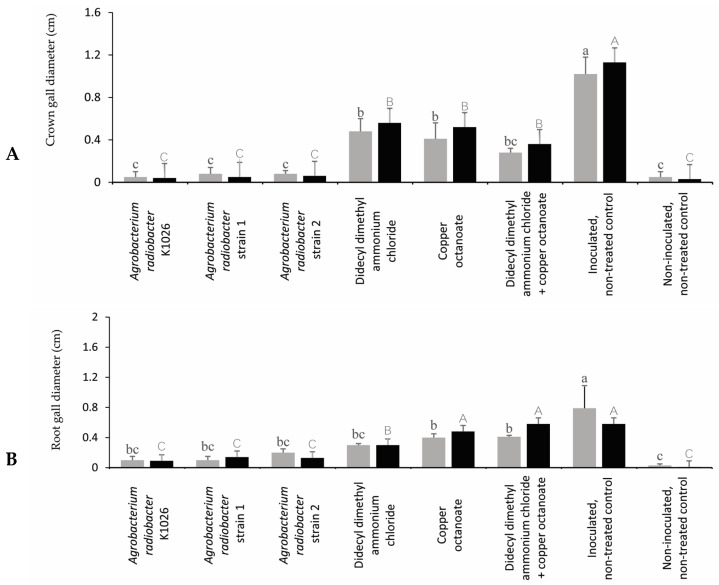
Crown (**A**) and root gall (**B**) diameters (mean ± SE) of the rose plants per treatment in the greenhouse trials. Means followed by different lowercase (trial 1) and uppercase (trial 2) letters above the bar representing the significant differences (n = 10, *p* ≤ 0.05, one-way ANOVA with post hoc Fisher’s test with an α = 0.05).

**Figure 5:**
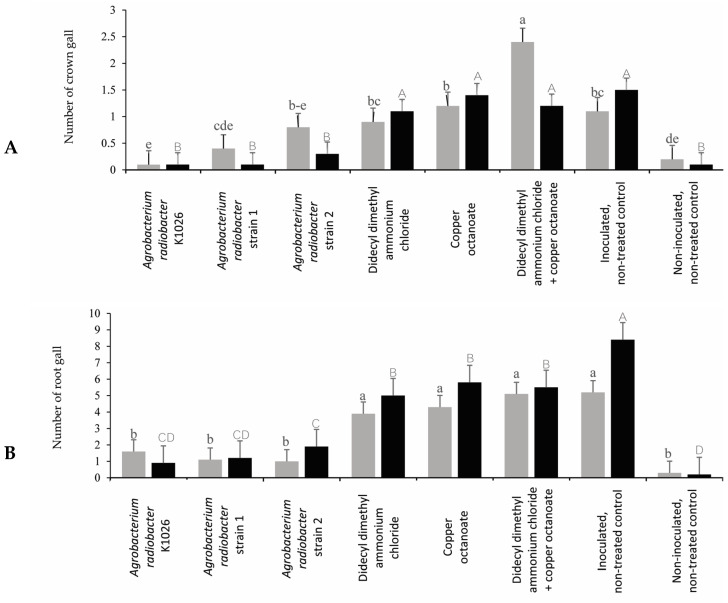
Number of crown (**A**) and root (**B**) galls (mean ± SE) for the rose plants per treatment in the greenhouse trials. Means followed by different lowercase (trial 1) and uppercase (trial 2) letters above the bar represent significant differences (n = 10, *p* ≤ 0.05, one-way ANOVA with post hoc the Fisher’s test the with an α = 0.05).

In trial 1, the *A. radiobacter* strain K1026 and *A. radiobacter* strains 1 and 2 reduced the number and diameter of crown galls compared to the inoculated, non-treated control (Figure 4 and Figure 5). In trial 1, the *A. radiobacter* strain K1026 and *A. radiobacter* strains 1 and 2 reduced the number of root galls; however, the chemical product treatments (didecyl dimethyl ammonium chloride, copper octanoate, and didecyl dimethyl ammonium chloride + copper octanoate) were not significantly different from the inoculated, non-treated control (Figure 5). In trial 2, all of the treatments showed a significant reduction in the number of crown galls. Moreover, the *A. radiobacter* strain K1026 and *A. radiobacter* strains 1 and 2 were more effective in reducing the crown gall diameters than the other treatments in trial 2. In both trials, the *A. radiobacter* strain K1026 and *A. radiobacter* strains 1 and 2 significantly reduced the number of root galls and root gall diameters compared to the chemical products and the inoculated, non-treated control (Figure 4).

**Figure 6:**
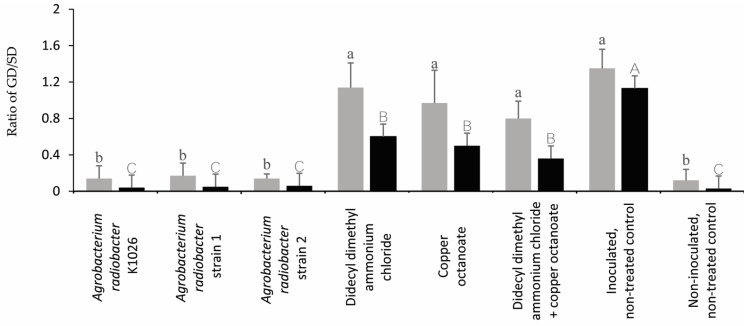
Effect of the treatments on the crown gall diameter to the stem diameter (GD/SD) ratio. Means followed by different lowercase (trial 1) and uppercase (trial 2) letters above the bar represent significant differences (n ₌ 10, *p* ≤ 0.05, one-way ANOVA with post hoc Fisher’s test with an α ₌ 0.05).

In trial 1, the plants treated with the *A. radiobacter* strain K1026 and *A. radiobacter* strains 1 and 2 resulted in a significant reduction in the GD/SD ratio compared to the didecyl dimethyl ammonium chloride, copper octanoate, didecyl dimethyl ammonium chloride + copper octanoate, and the inoculated, non-treated control (Figure 6). In trial 2, all of the treatments significantly reduced the GD/SD ratio compared to the inoculated, non-treated control (Figure 6, Supplementary Figure S1).

**Figure 7:**
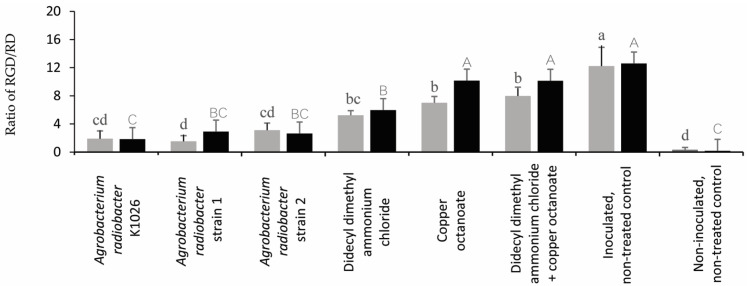
The effect of the treatments on the root gall diameter to the root diameter (RGD/RD) ratio. Means followed by different lowercase (trial 1, representing the gray color) and uppercase (trial 2, representing the black color) letters above the bar represent significant differences (n = 10, *p* ≤ 0.05, one-way ANOVA with post hoc Fisher’s test with an α = 0.05).

In trial 1, all of the treatments significantly reduced the RGD/RD ratio compared to the inoculated, non-treated control (Figure 7). However, the plants treated with the *A. radiobacter* strain K1026, *A. radiobacter* strains 1 and 2, and didecyl dimethyl ammonium chloride had a significant reduction in the RGD/RD ratio compared to the copper octanoate, didecyl dimethyl ammonium chloride + copper octanoate, and inoculated, non-treated control in trial 2 (Figure 7).

There were no differences in the plant height and width increments among the chemical and biological control products and the inoculated and non-inoculated rose plants in trial 1 (Table 2).

In trial 2, the plants treated with the *A. radiobacter* strain K1026 and didecyl dimethyl ammonium chloride had a significantly greater height increase compared to the inoculated, non-treated control. The *A. radiobacter* strains 1 and 2 and the inoculated, non-treated control had the lowest height increase, and were similar. There were no significant differences in the plant width among the treated and non-treated rose plants in trial 2. In trial 1, the non-inoculated, non-treated control and the *A. radiobacter* strain 2-treated plants had the greatest total fresh weight by the end of the trial (Table 2). The non-inoculated, non-treated control, *A. radiobacter* strains 1 and 2, and didecyl dimethyl ammonium chloride + copper octanoate had the greatest root fresh weight in trial 1. In trial 2, all of the treatments had significantly lower total and root fresh weights compared to the non-inoculated, non-treated control (Table 2). There were no significant differences in defoliation among the treatments in trial 1 (Table 3).

Didecyl dimethyl ammonium chloride had a significantly higher rate of defoliation in trial 2. In trial 1, all of the treatments had a significantly lower rate of chlorophyll compared to the non-inoculated, non-treated control. However, there was no significant difference in the chlorophyll content among the treatments in trial 2. The plant growth parameters were correlated with the number and diameter of root and crown galls in both trials. Nevertheless, no strong correlation was observed (Supplementary Tables S1 and S2).

The crown gall and root gall incidences were the highest for the inoculated, non-treated plants, with 100% in both trials. The plants treated with the *A. radiobacter* strain K1026 and *A. radiobacter* strains 1 and 2 reduced the crown gall incidence compared to the inoculated, non-treated control plants in both trials. Moreover, in both trials, the *A. radiobacter* strain K1026 and *A. radiobacter* strain 1 treatments decreased the root gall incidence compared with the inoculated, non-treated control plants. The rose plants treated with the *A. radiobacter* strain K1026 and *A. radiobacter* strain 1 had the lowest crown and root gall incidence in both trials. However, the didecyl dimethyl ammonium chloride + copper octanoate had no difference in crown and root gall incidence compared to the inoculated, non-treated control plants (Table 4).

### 3.4. Quantification of Agrobacterium tumefaciens from Rose Roots Using qPCR

Preliminary real-time PCR was run with a pure positive control of *A. tumefaciens*, which aimed to detect any nonspecific amplification products or primer dimers. A melting curve analysis from those runs could reveal the presence of such products at annealing temperatures below 55 °C or with shorter amplification times. The specific melting peak was detected at 83.5–84 °C.

A standard curve was constructed by plotting the known concentrations of pure *A. tumefaciens* DNA against the Ct values obtained from the real-time PCR (Supplementary Figure S2).

To validate the real-time PCR reproducibility, triplicate PCR amplifications were conducted using pathogen DNA from three different preparations. Highly reproducible Ct values with very small standard deviations were observed, yielding a linear regression coefficient (R2) of 0.999 for the standard curve. Within this standard curve, approximately four-cycle differences in Ct represented each 10-fold difference in the initial DNA amounts. Under the PCR conditions tested, the minimum starting quantity of the pathogen DNA that could be accurately quantified in our assays was 22.6 fg/µL, which corresponded to a Ct value of 36.33 ± 0.58. Threshold values suitable for quantification were established as a Ct of 37 (equivalent to 22.6 fg/µL of DNA according to the standard curve), an RFU of 1000, and an a-d(RFU)/dT of 200.

**Figure 8:**
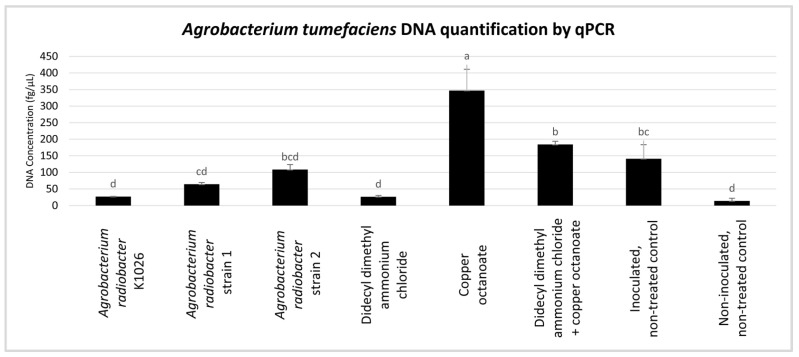
*Agrobacterium tumefaciens* DNA quantification in the roots of rose. The letters above the bar represent significant differences (n = 10, *p* ≤ 0.05, one-way ANOVA with post hoc Fisher’s test with an α ₌ 0.05).

To analyze the effect of the *A. radiobacter* strain K1026, *A. radiobacter* strains 1 and strain 2, didecyl dimethyl ammonium chloride, copper octanoate, and a combination of didecyl dimethyl ammonium chloride + copper octanoate on *A. tumefaciens*, the transcription levels of the *chvE* gene of *A. tumefaciens* were measured. Bacterial DNA was quantified in the plant roots using real-time PCR after the termination of trial 2 (Figure 8).

Copper octanoate showed the highest *A. tumefaciens* DNA quantity at 347.12 fg/µL, while the non-inoculated, non-treated control had the lowest at 13.98 fg/µL. The *A. tumefaciens* DNA quantity in didecyl dimethyl ammonium chloride + copper octanoate was 184.15 fg/µL; in the inoculated, non-treated control, it was 141.34 fg/µL; in *A. radiobacter* strain 2, it was 108.33 fg/µL; in *A. radiobacter* strain 1, it was 64.25 fg/µL, in the *A. radiobacter* strain K1026, it was 27.02 fg/µL; in the didecyl dimethyl ammonium chloride, it was 26.48 fg/µL. Low values were discarded from the analyses based on the threshold values and the limits of detection considered. Based on the result, the transcription level of the *chvE* gene was significantly reduced in the *A. tumefaciens* treated with the *A. radiobacter* strain K1026 and didecyl dimethyl ammonium chloride in the rose plants (Figure 8).

## 4. Discussion

*Agrobacterium tumefaciens* can infect a wide range of host plant species, including several woody ornamental plants. This leads to substantial annual losses for growers worldwide due to the unsellable nursery stock, reduced productivity from galled trees, and increased vulnerability of the infected plants to other pathogens and environmental stresses. [7,70,71,72,73,74,75,76,77,78,79,80,81,82]. Therefore, it is essential to be able to effectively treat and control crown gall. Identification based on phenotypic, biochemical, and molecular analyses showed that the bacteria was classified as the *Agrobacterium tumefaciens* biovar 1 species complex, which has the ability to utilize carbon in an agrocinopine-type manner [24,71,72,73,74].

As is known, biological control agents are successfully used to manage crown gall disease [11,75,76,77]. The *A. radiobacter* strain K1026 and *A. radiobacter* strains 1 and 2 were the most effective compared to the other treatments in reducing the number of root and crown galls, the diameter of the root and crown galls, and the GD/SD ratio in the current study. The *A. radiobacter* strains K84 and K1026 have already been reported to be effective in controlling crown gall diseases on different host plants [78,79]. Recently, the non-pathogenic strain of *A. vitis* ARK-1 controlled crown gall on apple (*Malus pumila*), Japanese pear (*Pyrus pyrifolia*), and peach (*Prunus persica*). The effectiveness of the strain ARK-1 treatment was calculated as an integrated risk ratio using the following formula: the proportion of plants developing tumors with the antagonist treatment divided by the proportion of plants developing tumors with the water treatment. The results showed the integrated risk ratio after the treatment with the ARK-1 strain to be 0.38 for apple crown gall, 0.16 for Japanese pear crown gall, and 0.20 for peach crown gall, indicating that the disease incidence was significantly reduced by the ARK-1 treatment [80]. Jones and Kerr [52] researched the biological control of crown gall on almonds (*Prunus dulcis*). The plants treated with the *A. radiobacter* strains K84 and K1026 were observed in 20% and 27% of the plants with galls, respectively. Similarly, *A. radiobacter* strain K1026 completely prevented gall formation on almond trees [81]. The presented study evaluated the crown gall incidence of *A. radiobacter* strains 1 and 2-treated and K1026-treated rose plants at 20%, 50%, and 10% in trial 1 and 10%, 30%, and 10% in trial 2, respectively. Moreover, Gupta et al. [75] reported that peach (*A. persica*) and cherry (*Prunus avium*) treated with *A. radiobacter* strains showed small crown gall diameters. New and Kerr [67] hypothesized that trees only become galled when there is a high ratio of pathogens to non-pathogens in the surrounding soil. This is supported by the evidence that *A. radiobacter* strains 1 and 2 and the *A. radiobacter* strain K1026 inhibited gall induction in the rose plants considered in the current study.

Didecyl dimethyl ammonium chloride causes membrane disruption, preventing pathogen resistance buildup and enabling broad-spectrum control [78]. Nguyen et al. [82] reported that the performance of QAC-2 containing a mixture of 6.51% of alkyl dimethyl benzyl ammonium chloride, 3.255% of didecyl dimethyl ammonium chloride, 3.255% of octyl decyl dimethyl ammonium chloride, and 8.68% of dioctyl dimethyl ammonium chloride as a sanitizer for pruning equipment contaminated with *Pseudomonas savastanoi* pv. *savastanoi* was effective in reducing knot formation on pruned trees. The application of didecyl dimethyl ammonium chloride as a sanitizer for pruning equipment has been found to be an effective treatment for several fungal and bacterial diseases [83,84,85,86,87,88]. In the current study, the drench application of didecyl dimethyl ammonium chloride was not found to be more effective in controlling crown gall disease; however, it can be a beneficial application for sanitizers for pruning equipment to prevent transferring bacterial inoculum to another plant.

Copper-based compounds lead to the denaturation of structural and enzymatic proteins and alter membrane semi-permeability [89]. There are conflicting reports on the effectiveness of copper treatments in controlling crown gall. Some studies have claimed that copper oxychloride is ineffective in controlling crown gall [44,90,91]. On the other hand, Utkhede and Smit [43] stated that, when the seedlings were treated with root-dip treatments of 2.5 and 5.0 g ai/L of copper oxychloride, the percentage of infection with crown gall was reduced. However, these treatments were phytotoxic on young apple trees, as measured by the percentage of dead trees and the reduced shoot growth. In the current study, copper octanoate reduced the number of crown and root galls and crown and root gall diameters compared to the inoculated, non-treated control. Additionally, the phytotoxicity was not observed on the treated rose plants.

A previous study suggested using didecyl dimethyl ammonium chloride as a sanitizer for the pruning process and copper compounds as a spray application after pruning [79,90]. In the current study, the treatment of didecyl dimethyl ammonium chloride + copper octanoate was used as a drench application, which decreased the crown gall diameter compared to the inoculated, non-treated control in trial 1. However, it was not as effective as the biological product treatments.

Currently, PCR- and qPCR-based methods are used to evaluate the effect of treatments or applications against plant diseases [11,56,92,93,94]. For trial 2, the qPCR quantification analysis was conducted to monitor the efficacy of chemical and biological products in the control of virulence gene expression from the tumor-inducing plasmid. Based on the number of root galls and RDG/RD observation, the plants treated with the *A. radiobacter* strain K1026 and *A. radiobacter* strains 1 and 2 had the lowest number of root galls in trial 2. Additionally, the *A. radiobacter* strain K1026, *A. radiobacter* strains 1 and 2, and didecyl dimethyl ammonium chloride reduced the ratio of RGD/RD. According to the qPCR analysis, the quantification of *A. tumefaciens* in the rose plants treated with the *A. radiobacter* strain K1026 and didecyl dimethyl ammonium chloride reduced transcription of the *chvE* gene in the rose plants. The results of the root galls and RGD/RD observations and the qPCR analysis were found to be nearly similar for the *A. radiobacter* strain K1026, *A. radiobacter* strains 1 and 2, and didecyl dimethyl ammonium chloride, and was effective against *A. tumefaciens*.

In this study, when the plants were visually monitored for the presence of crown and root galls caused by *A. tumefaciens* before setting up the experiments, no symptoms were detected. In both trials, the plants were inoculated with the same amount of solution (150 mL/plant) and the same bacterial solution concentration (1 × 10^9^ CFU mL^−1^). However, we observed crown and root galls on non-inoculated, non-treated control plants at the end of the experiments. It might be possible that the pathogen could be in the plant even if no symptoms were observed with the visual assessment of the plants. That can be a reason to observe gall formation caused by *A. tumefaciens* on non-inoculated, non-treated control plants. Additionally, it can explain the higher number of crown and root galls and diameters in the treated plants compared to the control plants, and the difference between trials 1 and 2. This suggests that using molecular tools to detect the pathogen can be more reliable before setting up the study for future experiments.

In conclusion, the management of this pathogen in nurseries poses a challenge due to the disease observation requirements and epidemiology. There are different ways suggested to manage bacterial diseases, including implementing sanitary and cultural practices. One effective strategy is to use biological control products such as the *A. radiobacter* strain K1026 and *A. radiobacter* strains 1 and 2 to control crown gall disease. This current study was able to identify and compare the effectiveness of biological controls and some chemical products for the management of crown gall disease. The findings revealed promising outcomes associated with the application of biological control products. Nonetheless, these results prepare the basis for future studies, which can be further tested in different combinations and rates.

## Figures and Tables

**Table 1:** Active ingredients and application rates of chemical and biological products used for this study.

Treatment	Application Rate
*Agrobacterium radiobacter* strain K1026	0.623 g·L^−1^
*Agrobacterium radiobacter* strain 1	1.2 × 10^9^ CFU mL^−1^
*Agrobacterium radiobacter* strain 2	1.2 × 10^9^ CFU mL^−1^
Didecyl dimethyl ammonium chloride	4 mL·L^−1^
Copper octanoate	10 mL·L^−1^
Didecyl dimethyl ammonium chloride + copper octanoate	2 mL·L^−1^ + 5 mL·L^−1^

**Table 2:** Total plant fresh weight, fresh root weight, height, and width increments (mean ± SE) of the rose plants treated with chemical and biological products in the greenhouse trials.

Treatment	Height Increase (cm)		Width Increase (cm)		Total Fresh Weight (g)		Root Fresh Weight (g)	
	Trial 1	Trial 2	Trial 1	Trial 2	Trial 1	Trial 2	Trial 1	Trial 2
*Agrobacterium radiobacter* strain K1026	30.73 ± 2.29 a*	33.65 ± 0.72 a	11.68 ± 1.27 a	12.05 ± 0.68 a	34.01 ± 2.83 bc	30.50 ± 2.14 bc	17.29 ± 1.98 bc	15.10 ± 1.38 bc
*Agrobacterium radiobacter* strain 1	21.84 ± 3.30 a	25.10 ± 1.05 bcd	12.45 ± 1.78 a	13.90 ± 0.62 a	34.01 ± 2.83 bc	28.90 ± 2.94 bc	18.42 ± 1.70 bc	16.10 ± 2.20 bc
*Agrobacterium radiobacter* strain 2	22.10 ± 2.54 a	24.60 ± 1.92 cd	16.0 ± 1.52 a	13.90 ± 0.59 a	42.52 ± 5.66 ab	36.22 ± 1.94 b	22.39 ± 2.83 ab	18.78 ± 1.76 b
Didecyl dimethyl ammonium chloride	28.19 ± 2.79 a	29.40 ± 1.25 ab	14.22 ± 2.03 a	12.63 ± 0.68 a	25.51 ± 2.83 c	23.95 ± 2.30 c	13.89 ± 1.13 c	11.40 ± 1.25 c
Copper octanoate	25.91 ± 3.30 a	27.45 ± 1.62 bc	15.24 ± 2.29 a	13.40 ± 0.96 a	34.01 ± 5.66 bc	30.13 ± 2.27 bc	18.42 ± 3.40 bc	15.19 ± 1.60 bc
Didecyl dimethyl ammonium chloride + copper octanoate	25.91 ± 2.29 a	27.10 ± 1.73 bc	10.41 ± 1.02 a	11.15 ± 0.72 a	39.68 ± 2.83 b	33.30 ± 4.39 b	19.27 ± 1.41 abc	17.90 ± 2.33 b
Inoculated, non-treated control	18.29 ± 2.29 a	22.0 ± 1.75 d	11.43 ± 1.27 a	12.85 ± 0.81 a	31.18 ± 2.83 bc	28.85 ± 3.9 bc	17.57 ± 1.41 bc	15.25 ± 1.90 bc
Non-inoculated, non-treated control	25.15 ± 3.30 a	28.65 ± 2.32 bc	12.45 ± 1.52 a	13.05 ± 0.89 a	51.02 ± 5.66 a	46.89 ± 2.05 a	24.66 ± 1.98 a	25.06 ± 1.10 a
*p* value	0.0777	0.0002	0.155	0.1676	0.001	0.0001	0.0233	0.0001
*F* value	1.93	4.72	1.58	1.54	4.14	5.29	2.5	4.97

* Treatment means followed by different lowercase letters in the column denote a significant difference at *p* ≤ 0.05. The values are the means per plant for ten single-plant replicates. A one-way analysis of variance was used to evaluate treatment effects. When the effects were significant, Fisher’s least significant difference test was used for the mean comparisons with α = 0.05. The same letters mean no statistical difference.

**Table 3:** Defoliation and chlorophyll content (SPAD value) (mean ± SE) of the rose plants treated with chemical and biological control products in the greenhouse trials.

Treatment	Defoliation (%)		Chlorophyll Content (SPAD Value)	
	Trial 1	Trial 2	Trial 1	Trial 2
*Agrobacterium radiobacter* strain K1026	20 ± 4 a*	21 ± 5 b	42.72 ± 1.26 bc	45.54 ± 1.33 a
*Agrobacterium radiobacter* strain 1	14 ± 4 a	13 ± 5 b	43.60 ± 1.37 bc	44.43 ± 1.36 a
*Agrobacterium radiobacter* strain 2	27 ± 11 a	30 ± 13 b	40.90 ± 2.51 c	43.61 ± 1.68 a
Didecyl dimethyl ammonium chloride	44 ± 9 a	47 ± 10 a	44.63 ± 1.56 abc	47.39 ± 1.44 a
Copper octanoate	31 ± 12 a	36 ± 13 b	40.88 ± 3.34 c	42.94 ± 2.93 a
Didecyl dimethyl ammonium chloride + copper octanoate	17 ± 9 a	19 ± 9 b	43.33 ± 1.24 bc	44.88 ± 1.28 a
Inoculated, non-treated control	29 ± 10 a	27 ± 9 b	47.10 ± 2.15 ab	46.17 ± 2.13 a
Non-inoculated, non-treated control	11 ± 2 a	12 ± 3 b	49.44 ± 1.94 a	47.86 ± 1.66 a
*p* value	0.0541	0.0037	0.0476	0.4713
*F* value	2.11	3.42	2.17	0.95

* Treatment means followed by different lowercase letters in the column denote a significant difference at *p* ≤ 0.05. The values are the means per plant for ten single-plant replicates. A one-way analysis of variance was used to evaluate treatment effects. When the effects were significant, Fisher’s least significant difference test was used for the mean comparisons with α = 0.05. The same letters mean no statistical difference.

**Table 4:** Efficacy of the chemical and biological control products in the prevention of crown and root galls in rose caused by *Agrobacterium tumefaciens*.

Treatment	Crown Gall Incidence (%)		Root Gall Incidence (%)	
	Trial 1	Trial 2	Trial 1	Trial 2
*Agrobacterium radiobacter* strain K1026	10	10	30	30
*Agrobacterium radiobacter* strain 1	20	10	30	40
*Agrobacterium radiobacter* strain 2	50	30	60	50
Didecyl dimethyl ammonium chloride	90	90	90	100
Copper octanoate	80	100	90	100
Didecyl dimethyl ammonium chloride + copper octanoate	100	100	100	100
Inoculated, non-treated	100	100	100	100
Non-inoculated, non-treated	10	10	10	10

## Data Availability

Data are unavailable due to privacy or ethical restrictions.

## Supplementary Materials

The following supporting information can be downloaded at: https://www.mdpi.com/article/10.3390/pathogens13080708/s1. Table S1. Coefficients of linear correlation (r) for the relationship between plant growth parameters and the number of root and crown galls. Table S2. Coefficients of linear correlation (*r*) for the relationship between plant growth parameters and the root and crown gall diameters. Figure S1. (A) measure the crown gall diameter (indicated by the gray arrow) and the stem gall diameter (indicated by the black arrow) using the caliper to calculate GD/SD, (B) measure the root gall diameter (indicated by the gray arrow) and the root diameter (indicated by the black arrow) using the caliper to calculate RGD/RD. Figure S2. The qPCR amplification of 10-fold dilutions (triplicate) and the standard curve graph used for absolute quantification of *Agrobacterium tumefaciens*.
