# Supplementary material for: Evaluation of Chemical and Biological Products for Control of Crown Gall on Rose
Source: Pathogens. 2024 Aug 21;13(8):708. doi: 10.3390/pathogens13080708 (PMC11357299; doi:10.3390/pathogens13080708)
Supplement: Supplementary file 1 [file pathogens-13-00708-s001.zip › pathogens-3131532-supplementary.pdf]

Supplementary material

Table S1. Coefficients of linear correlation (r) for the relationship between plant growth parameters and the number of root and crown galls.

**Parameter**	**Number of root gall**				**Number of crown gall**			
	Trial 1		Trial 2		Trial 1		Trial 2	
	R	*P* value	*r*	*P* value	*r*	*P* value	*r*	*P* value
Height increase	-0.19	0.80	-0.37	0.12	-0.10	0.36	-0.09	0.37
Width increase	-0.07	0.49	-0.06	0.54	-0.16	0.15	-0.02	0.79
Defoliation	0.03	0.73	0.19	0.088	0.11	0.32	0.11	0.32
Chlorophyll	-0.10	0.35	0.05	0.61	0.01	0.92	0.06	0.57
Total fresh weight	-0.18	0.10	-0.32	0.003	-0.12	0.25	-0.11	0.31
Root fresh weight	-0.18	0.09	-0.32	0.003	-0.14	0.20	-0.13	0.23

Table S2. Coefficients of linear correlation (*r*) for the relationship between plant growth parameters and the root and crown gall diameters.

**Parameter**	**Root gall diameter**				**Crown gall diameter**			
	Trial 1		Trial 2		Trial 1		Trial 2	
	R	*P* value	*r*	*P* value	*r*	*P* value	*r*	*P* value
Height increase	-0.15	0.16	-0.22	0.04	-0.18	0.10	-0.14	0.20
Width increase	-0.12	0.26	-0.21	0.05	-0.12	0.28	-0.01	0.90
Defoliation	0.02	0.79	0.02	0.85	0.16	0.13	0.13	0.24
Chlorophyll	0.19	0.08	-0.11	0.32	0.08	0.43	0.11	0.29
Total fresh weight	-0.10	0.34	-0.10	0.34	-0.18	0.10	-0.22	0.04
Root fresh weight	-0.08	0.43	-0.08	0.45	-0.14	0.20	-0.23	0.04

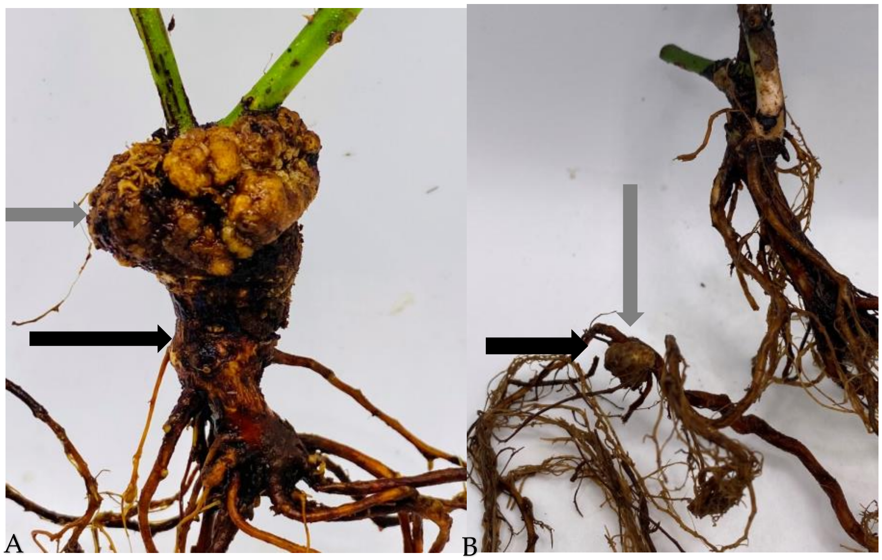

Figure S1. A: measure the crown gall diameter (indicated by the gray arrow) and the stem gall diameter (indicated by the black arrow) using the caliper to calculate GD/SD, B: measure the root gall diameter (indicated by the gray arrow) and the root diameter (indicated by the black arrow) using the caliper to calculate RGD/RD

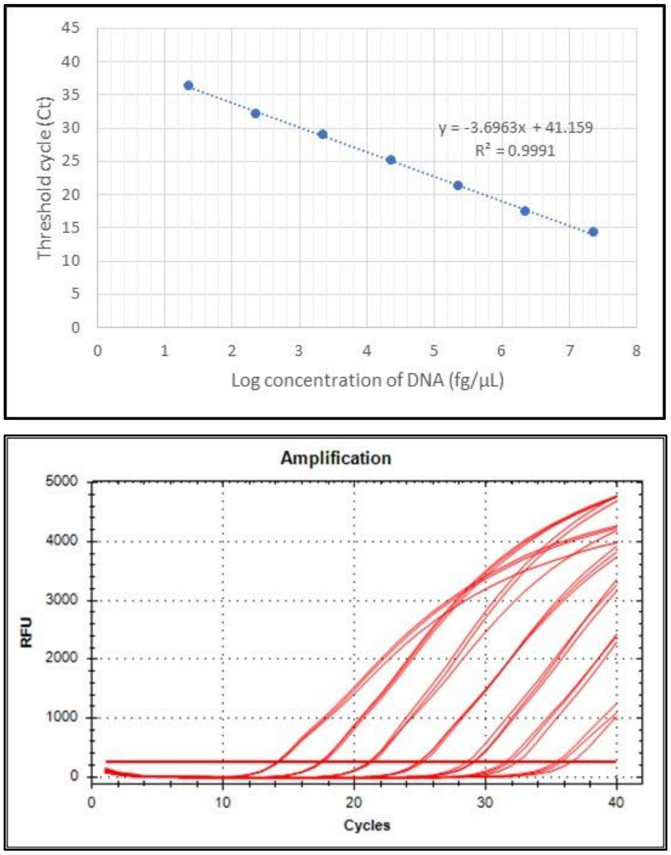

Figure S2. The qPCR amplification of 10-fold dilutions (triplicate) and the standard curve graph used for absolute quantification of *Agrobacterium tumefaciens*.
